# Dynamic changes in membrane lipid composition of leaves of winter wheat seedlings in response to PEG-induced water stress

**DOI:** 10.1186/s12870-020-2257-1

**Published:** 2020-02-21

**Authors:** Yajing Wang, Xinying Zhang, Guirong Huang, Fu Feng, Xiaoying Liu, Rui Guo, Fengxue Gu, Xiuli Zhong, Xurong Mei

**Affiliations:** Institute of Environment and Sustainable Development in Agriculture, Chinese Academy of Agricultural Sciences/State Engineering Laboratory of Efficient Water Use and Disaster Mitigation for Crops/Key Laboratory for Dryland Agriculture of Ministry of Agriculture, Beijing, 100081 China

**Keywords:** Phospholipids, Galactolipids, Photosynthesis, Lipid profiling, Lipid unsaturation

## Abstract

**Background:**

Membrane lipid composition associates closely with membrane stability and fluidity under water stress. In this study, lipidomic analyses based on electrospray ionization mass spectrometry (ESI-MS/MS) were carried out to explore dynamic changes of membrane lipids in term of molecular species caused by PEG (Polyethylene glycol-6000)-induced water stress in wheat seedlings.

**Results:**

Among the main phospholipids, phosphatidylcholine (PC), phosphatidylethanolamine (PE), and phosphatidylglycerol (PG) are primary degradation targets, and PC was degraded in the largest degree. Membrane ion leakage dramatically increased later than the significant reduction of these phospholipids, indicating that the loss of membrane integrity lagged behind severe phospholipid degradation. Monogalactosyldiacylglycerol (MGDG) increased firstly and decreased later, while digalactosyldiacylglycerol (DGDG) ratcheted up with stress. DGDG/MGDG increased after stress for 3 days, and unsaturation of DGDG was promoted with stress. Variation trends of galactolipids differed among molecular species. The time when MGDG (34:3), DGDG (34:3) began to decline approached to the time when non-stomatal limitation impaired photosynthesis. While the two predominant molecular species MGDG (36:6) and DGDG (36:6) began to decline later. So we speculated that MGDG (34:3), DGDG (34:3) might be key components in photosynthesis apparatus and participate in photosynthesis directly. While the two predominant molecular species, MGDG (36:6) and DGDG (36:6) might locate in thylakoid lipid bilayer matrix and play roles in stabilizing the membrane. The research provides new insights into the dynamic response of lipid metabolism to PEG-induced water stress.

**Conclusion:**

In wheat plants under water stress, the major molecular species of PC, PE and PG were degraded, MGDG and DGDG molecular species had differing degradation time courses.

## Background

Phospholipids are the main structural components of cellular membranes. Various abiotic stresses, such as drought, salt, and freezing, activate distinct phospholipases, and distinct isoforms of each phospholipase. Afterwards, the activated phospholipases preferentially hydrolyze membrane phospholipids [1], leading to differential degradation of these lipids. In *Arabidopsis thaliana* subjected to freezing stress, Phosphatidylcholine (PC) was substantially degraded, while phosphatidylethenolamine (PE), phosphatidylglycerol (PG), phosphatidylserine (PS), and phosphatidylinositol (PI) were degraded relatively less [2]. Decreases in absolute content of many lipids occurred after stresses, but the proportion of different lipids varies [3, 4]. Moreover, the composition of molecular species of each lipid class, which shares a head group but differs in chain length and double-bond number, also change in response to stresses [5]. Due to the shape of each lipid class, PC, PG and PS tend to form stable bilayer structure, while others, such as PE, are apt to form unstable non-lamellar structure [2, 6]. Unsaturated lipids play an important role in membrane stabilization in plants [7, 8]. Increased fatty acid desaturation via over expression of ω-3 desaturases in tobacco resulted in enhanced tolerance under both drought and salt stresses [9]. Lipid composition thus has an important influence on the integrity of cellular membrane and on the intrinsic-membrane protein activities under stresses [10–12].

Thus maintenance of the structural integrity of cellular membrane, which is essential to maintain metabolic homeostasis [13], is a prerequisite for survival during adverse environmental conditions [14, 15]. The main targets under environmental stresses, membrane lipids, whose metabolism plays a crucial role in membrane stabilization under adverse environmental conditions [7, 16]. Plants have evolved the ability to positively modulate lipid composition to adapt to environmental stresses. Lipid unsaturation degree increases under stresses, as found in chickpea (*Cicer arietinum*) under low temperature [17], *Arabidopsis thaliana* under cold acclimation [2], wheat (*Triticum aestivum* L.) [18] and resurrection plant *Xerophata humilis (Bak)* under dehydration [4]. With increasing stress severity, however, passive degradation of a large amount of lipids occurs unavoidably, leading to the ultimate collapse of membrane integrity and drastic increased ion leakage from cells. Nevertheless, how membrane lipid components dynamically change in the process of stress development from mild to severe has been rarely reported to date.

Galactosylglycerides, monogalactosyl-diacylglycerol (MGDG) and digalactosyl-diacylglycerol (DGDG), are the main structural components of photosynthetic membranes, representing 70–80% of total lipids in the membranes of chloroplast and thylakoid [19, 20]. DGDG, which possesses a cylindrical shape, forms stable bilayer lamellar phase easily, while MGDG is more likely to form an unstable hexagonal phase II due to its conical shape [21, 22]. Modification of the two kinds of galactolipids could affect the biophysical properties of photosynthetic membranes and thus plays an important role in stress response in photosynthesis. Under stresses, plants tend to increase the absolute content of DGDG and the ratio of DGDG to MGDG to maintain the stability of the chloroplast membrane [7, 23]. Drought tolerant genotypes change more than the sensitive genotypes. This has been observed in several species, such as *Arabidopsis thaliana* [24], cowpea (*Vigna unguiculata* L.(Walp.)) [7], *Craterostigma plantagineum* [3], and maize (*Zea mays* L.) [25]. In recent years, research found that galactolipids not only establish the lipid bilayer in which the photosynthetic complexes are embedded, but also participate in photosynthesis light reactions [26, 27]. Galactolipids, thus are becoming a focus in research about adaptation mechanisms of plant photosynthesis. But how the galactolipid composition changes dynamically with stress severity aggravating remains unknown.

In most cases, conclusions on lipid degradation, conversion, and the modification of acyl chain unsaturation under stresses were based on single investigation. With increasing stress, the dynamic changes in lipid composition, which includes lipid molecular species in particular, remains unknown to date. In this study, lipidomic analyses based on electrospray ionization mass spectrometry (ESI-MS/MS) were carried out to explore dynamic changes of membrane lipids in term of molecular species caused by water stress in wheat leaves. Through the comprehensive, comparative and dynamic analysis, we found that PC, PE, and PG were primary degradation targets, with their main molecular species and total contents rapidly declining to the low levels within 2 d of stress, earlier than the dramatic increase in membrane ion leakage. Galactolipid molecular species differed in their changes with time. The time MGDG (34:3) and DGDG (34:3) began to decline was almost coincident with the time photosynthesis rate declined caused by non-stomatal limitation factors. This may imply distinct functions of these galactolipid molecular species.

## Results

### Cellular membrane ion leakage increased with time of water stress

**Fig. 1:**
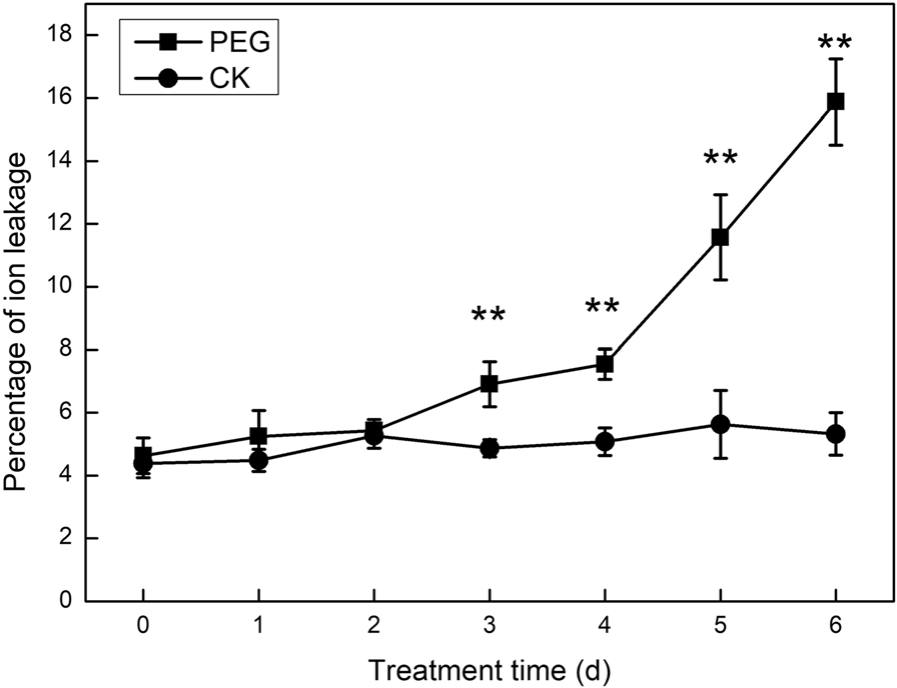
Changes of ion leakage with water stress time. Ion leakages in leaves were measured at each day from 0 to 6 days after treatment with PEG (PEG) and without PEG (CK). Values are means ± SE (*n* = 3). One asterisk (*) and two asterisks (**) represent significant differences between PEG and CK at *P* < 0.05 and *P* < 0.01 respectively

As well known, stress severity is determined by both stress intensity and stress time. During the 6 days treatment, PEG concentration was kept at 20% constantly. This means that drought severity the wheat leaves suffered increased over time. Correspondingly, the membrane ion leakage demonstrated an increasing trend with time. It became significantly higher than CK at the 3 days after stress, and dramatically increased after 4 days under water stress, reaching almost 3 times of that in CK (*P* < 0.01) on the 6th day (Fig. 1; Additional file 1). Ion leakage of cellular membrane relates closely to its integrity and stability. This variation curve thus indicated that cellular membrane suffered light injury 3–4 days after onset of such stress condition. Strikingly, severe damage to the integrity and stability of cellular membrane occurred 4 days after PEG treatment at this stress intensity.

### Phospholipid composition changes with time of water stress

Membrane phospholipids, crucial components of membrane skeleton, include PC, PE, PG, phosphatidylserine (PS) and phosphatidylinositol (PI). With water stress time proceeding, levels of the main phospholipids, especially in term of molecular species, were measured dynamically (Additional file 1).

**Fig. 2:**
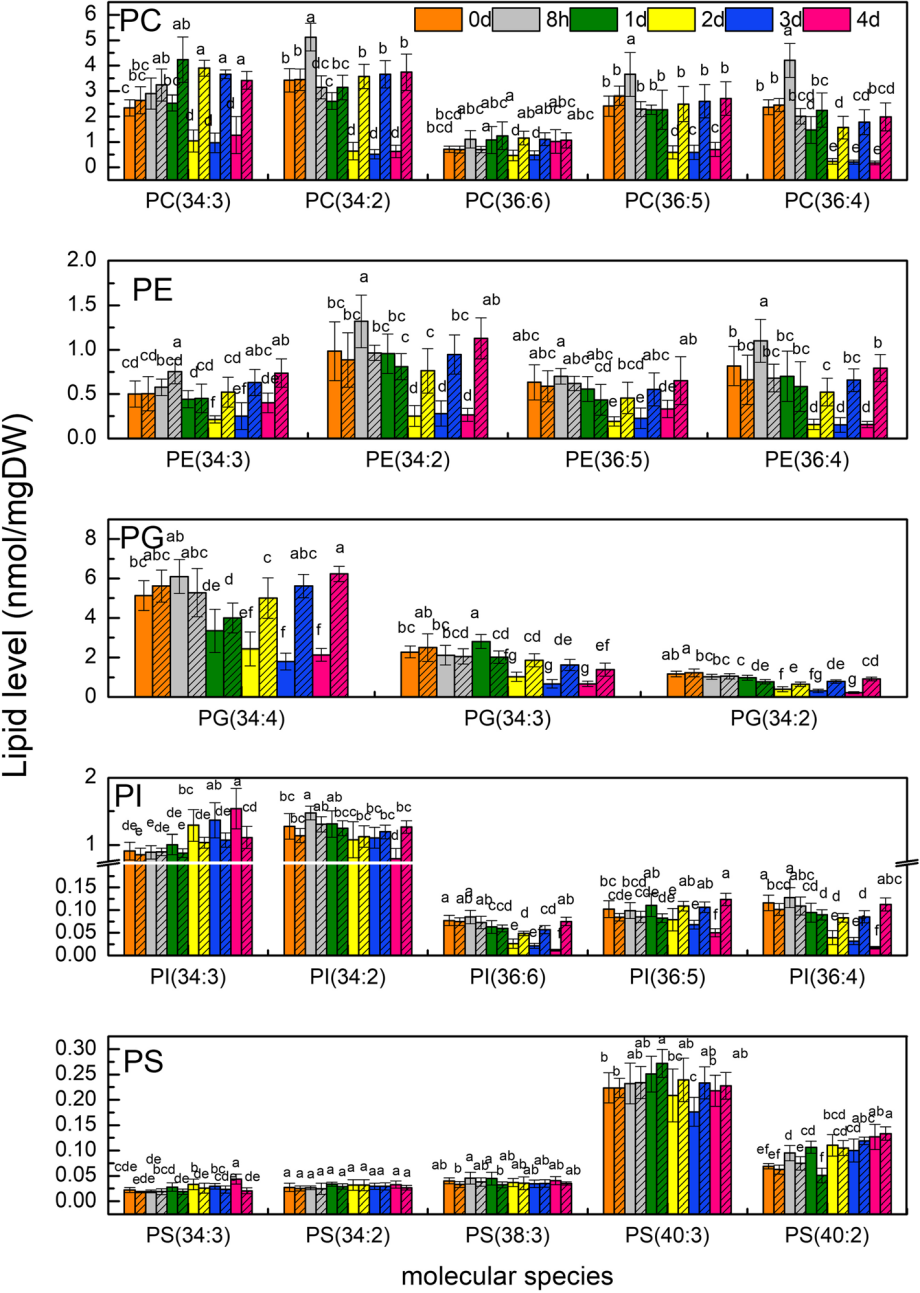
Phospholipid composition changes with water stress time. Phospholipid molecular species were determined in plants after treatment for 0 day, 8 h, 1 day, 2 days, 3 days, and 4 days under PEG (PEG, blank bars) and CK (without PEG, hatched bars) treatments. Different letters indicate significant difference (*P* < 0.05). Values are means ± SE (*n* = 4 to 6)

PC, PE, PG were main phospholipids in plant tissue. In the wheat, PC (34:3), PC (34:2), PC (36:5), PC (36:4), PC (36:6), were the main PC molecular species, making up 90% of total PC content. The former 4 molecular species increased during the first 8 h under PEG treatment, and decreased sharply after that (Fig. 2). PC (36:6) showed lower content after treatment for 2 to 3 days, while after 4 days under treatment, it showed no significant difference between plants under PEG and CK (without PEG) treatments.

PE (34:3), PE (34:2), PE (36:5), and PE (36:4) were the main PE molecular species, making up 85% of total PE content. PE (34:3) and PE (36:5) kept stable within 1 day under stress, and decreased to a low level 2 days after PEG treatment. PE (34:2) and PE (36:4) increased slightly at the early stage and decreased after 8 h under stress.

PG (34:4), PG (34:3), PG (34:2) were the main PG molecular species, accounting for 90% of total PG content. The 3 molecular species kept stable firstly and decreased to a low level 2 days after PEG treatment, then remained unchanged in the following days.

**Fig. 3:**
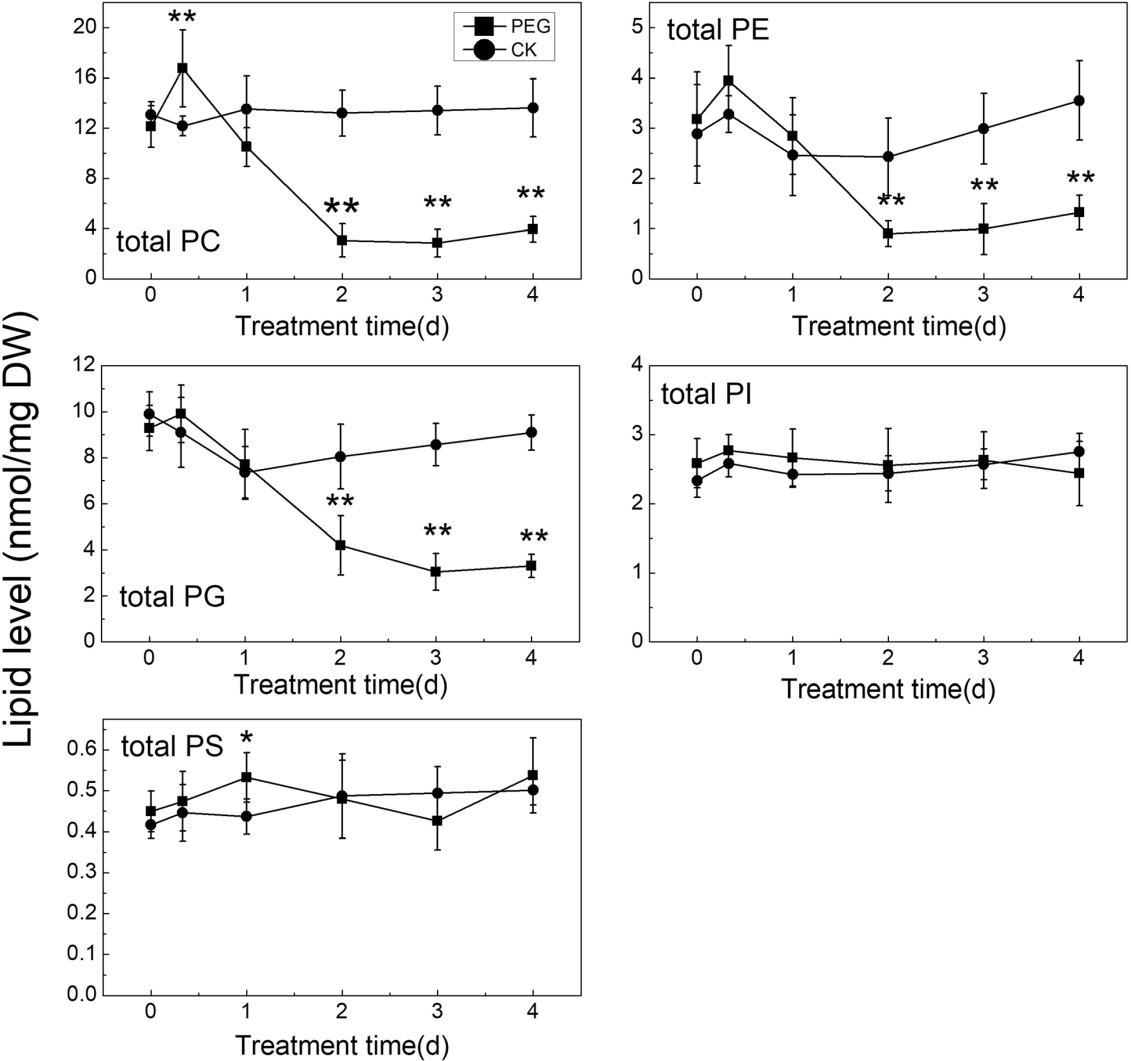
Total phospholipid changes with water stress time. * and ** indicate significant differences between plants under treatments with PEG (PEG) and without PEG (CK) at the same time points of determination at *P* < 0.05 and *P* < 0.01 respectively. Values are means ± SE (*n* = 4 to 6)

The content of total PC, PE, and PG, as well as that of their respective main molecular species demonstrated very similar trends with stress time (Fig. 3). The lipid levels slightly increased in the early stages, then rapidly declined to a low value 2 days after stress and kept nearly stable for the following 2 days. This indicated these phospholipids declined to the low levels earlier than the time when membrane ion leakage began to increase.

Total PI kept nearly stable for 4 days of PEG stress. No significant difference in total PI existed between plants under PEG treatment and CK. Different PI molecular species, however, presented diverse dynamic trends under treatment. PI (34:3) and PI (34:2) are the main PI molecular species in wheat leaves, which accounted for 80% of total PI. As shown in Fig. 2, PI (34:2) showed a decreased trend with stress time, being significantly lower than CK on the 4th day. In contrast, PI (34:3) showed an upward trend. Plus, other minor PI molecular species, PI (36:6), PI (36:5), PI (36:4), PI (36:3), and PI (36:2) showed downward trends.

PS (34:3), PS (34:2), PS (38:3), PS (40:3), PS (40:2) presented in Fig. 2 made up 80% of total PS. The total PS showed a fluctuating trend, and the most abundant molecular species PS (40:3) followed the trend apparently, PS (40:2) increased generally, while PS (34:2) and PS (38:3) kept almost stable with water stress continued. It deserves to be mentioned that differing from the 4 classes of phospholipids above, none of the main molecular species of PS showed a clear downward trend, implying that PS was not the main phospholipid degraded under water stress.

**Fig. 4:**
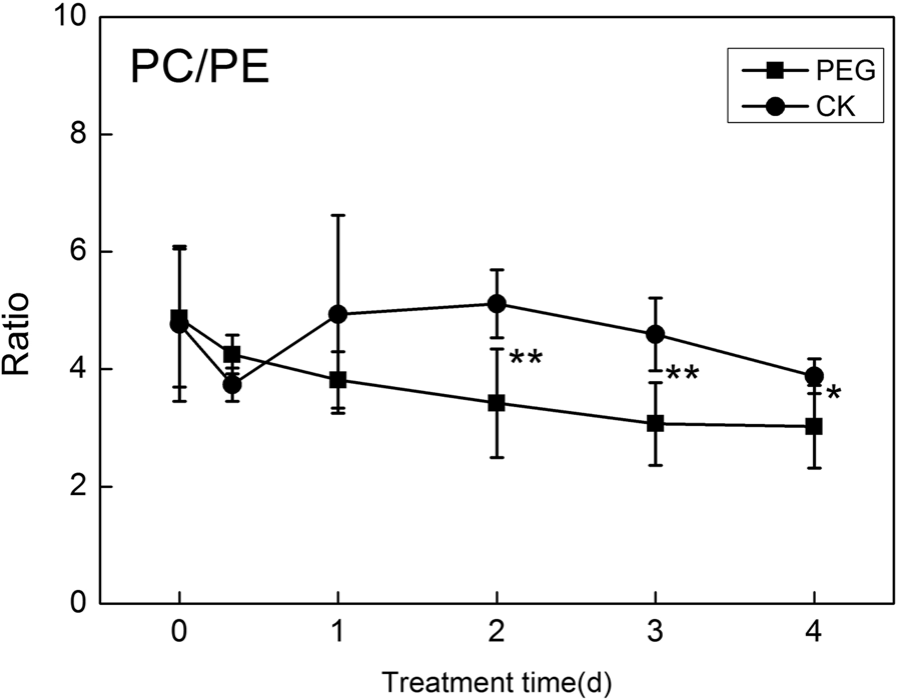
Changes of PC/PE with time of water stress. * and ** indicate significant differences between plants under treatments with PEG (PEG) and without PEG (CK) at the same time points of determination at *P* < 0.05 and *P* < 0.01 respectively. Values are means ± SE (*n* = 4 to 6)

The ratio of PC to PE in different molecular species under PEG treatment is consistent generally. PC/PE curve declined with stress time, and it was lower than that of CK significantly after 2 days under stress (Fig. 4). Apparently, the PC/PE falling down was earlier than the time when significant increase in membrane ion leakage occurred.

### DBI of phospholipids changes under water stress

**Fig. 5:**
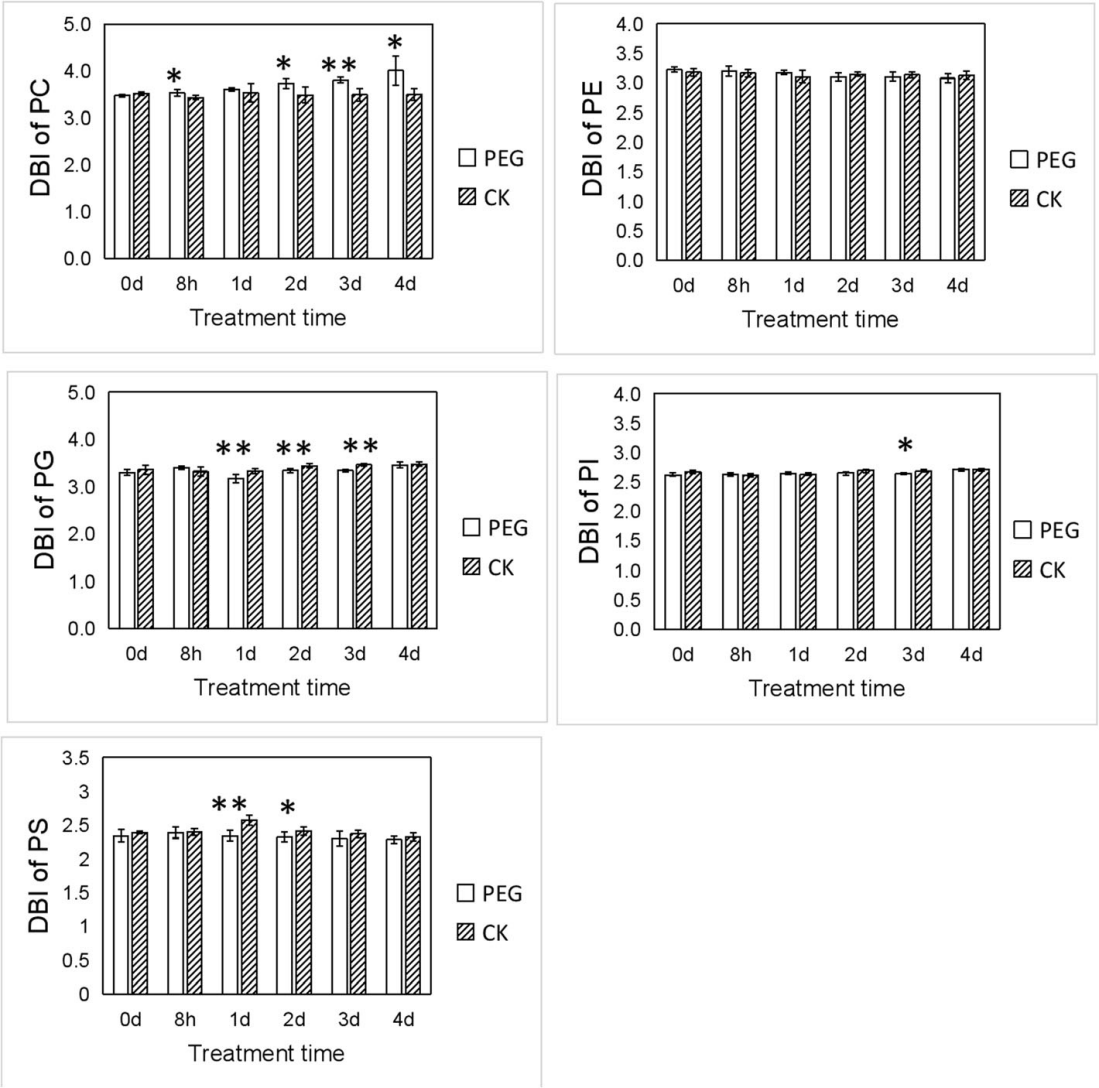
Changes in the DBI of phospholipids with water stress time. DBI = Σ(n × mol% lipid)/100, where n is the total number of double bonds in the two fatty acid chains of each glycerolpid molecule. * and ** indicate significant differences between plants under treatment with PEG (PEG) and without PEG (CK) at the same time points of determination at *P* < 0.05 and *P* < 0.01 respectively. Values are means ± SE (*n* = 4 to 6)

DBI is an important indicator for it is positively related to the fluidity of membrane lipids. As shown in Fig. 5 and Additional file 1, no significant difference was observed in PE. DBI of PC increased significantly after water stress for 8 h, and increased further in later days of stress apart from 1d. Differing from PC, DBI of PI, PG and PS showed decreases in DBI at different times significantly within 1 day to 4 days after stress. DBI of PS was 9.05% lower than that of CK after 1 day under stress. The results above indicated that PC was the only phospholipid that responded to water stress by elevating unsaturation.

### Dynamic changes of photosynthetic parameters in response to water stress

**Fig. 6:**
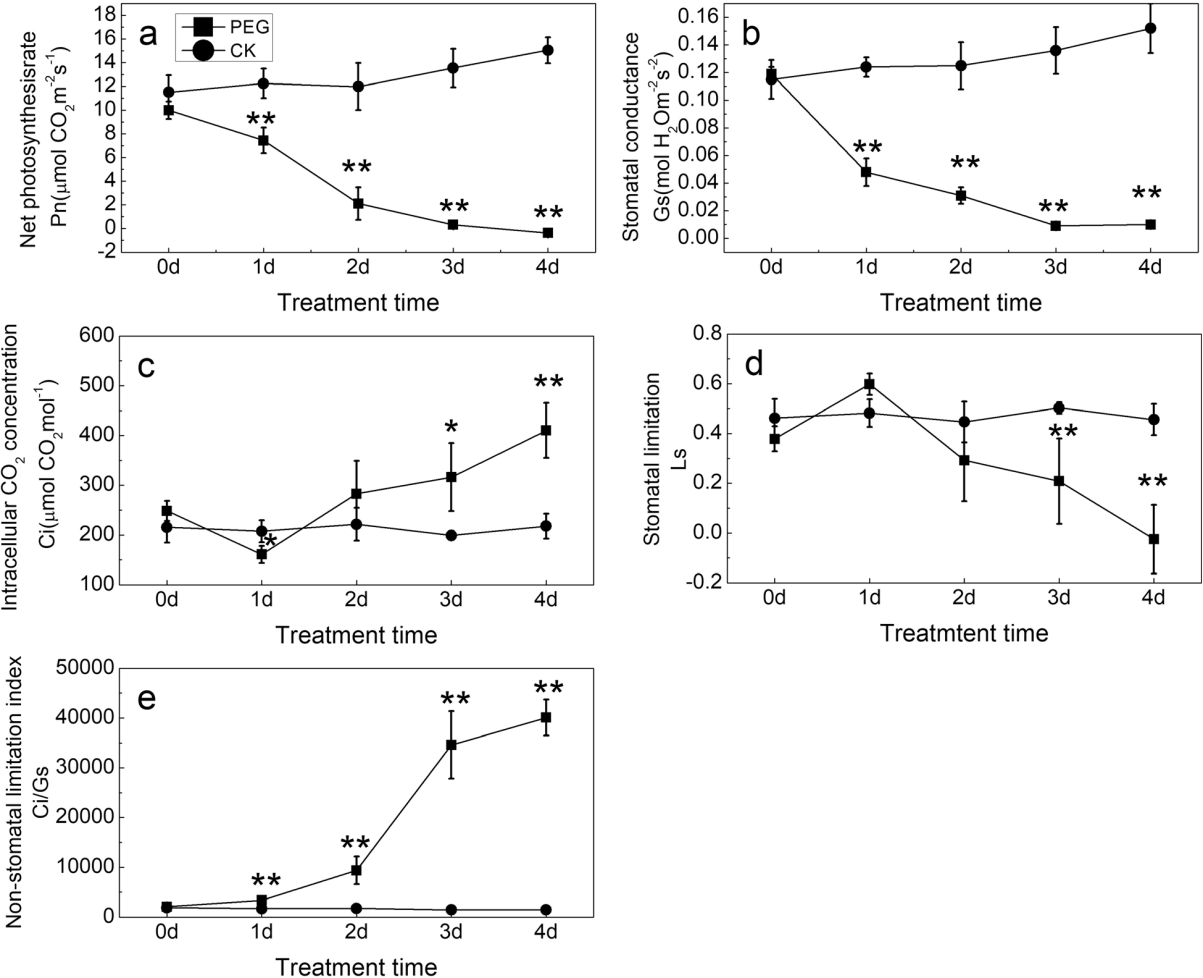
Changes in photosynthesis parameters of plants with water stress time. **a** net photosynthesis rate (Pn); **b** stomatal conductance (Gs); **c** intracellular CO_2_ concentration (Ci); **d** stomatal limitation index (Ls) =1-Ci/Ca, Ca = 400 μmol·mol^− 1^ here; **e** non-stomatal limitation index = Ci/G_s_. * and ** indicate significant differences between plants under treatment with PEG (PEG) and without PEG (CK) at the same time points of determination at *P* < 0.05 and *P* < 0.01 respectively. Values are means ± SE (*n* = 3)

Under PEG treatment, net photosynthetic rate (Pn) decreased with stress time proceeding (Fig. 6a; Additional file 1), with a dramatic decline to 79% at 1 day after treatment. Similarly, the stomatal conductance (G_s_) also decreased over time, but the dramatic decrease occurred on the first day (Fig. 6b). Differing from the both, the intracellular CO_2_ concentration (Ci) declined within the first day only, and then, it increased with time in the following days (Fig. 6c). Remarkably, it was Ci increase, rather than Ci decrease, that accompanied Gs decline by after 1 day under stress. The results demonstrated that the decrease in Pn after 1 day under PEG stress mainly resulted from the decline of efficiency of photosynthetic apparatus, but not the decline in Gs. This was further supported by the variation in both stomatal limitation index (Ls) (Fig. 6d) and non-stomatal limitation index (Ci/Gs) (Fig. 6e) during stress period. Ls increase in the first day was consistent with Ci decrease, suggesting that it was mainly due to Gs reduction brought about Pn decrease. The following, Ls decrease with time indicated that Gs was not the main limiting factor for photosynthesis. Ci/Gs slightly increased in the first day, then rose rapidly in the following days, implying that the poor function of photosynthetic apparatus turned to be the predominant factor in limiting photosynthesis.

### Galactolipid composition changes with time of water stress

**Fig. 7:**
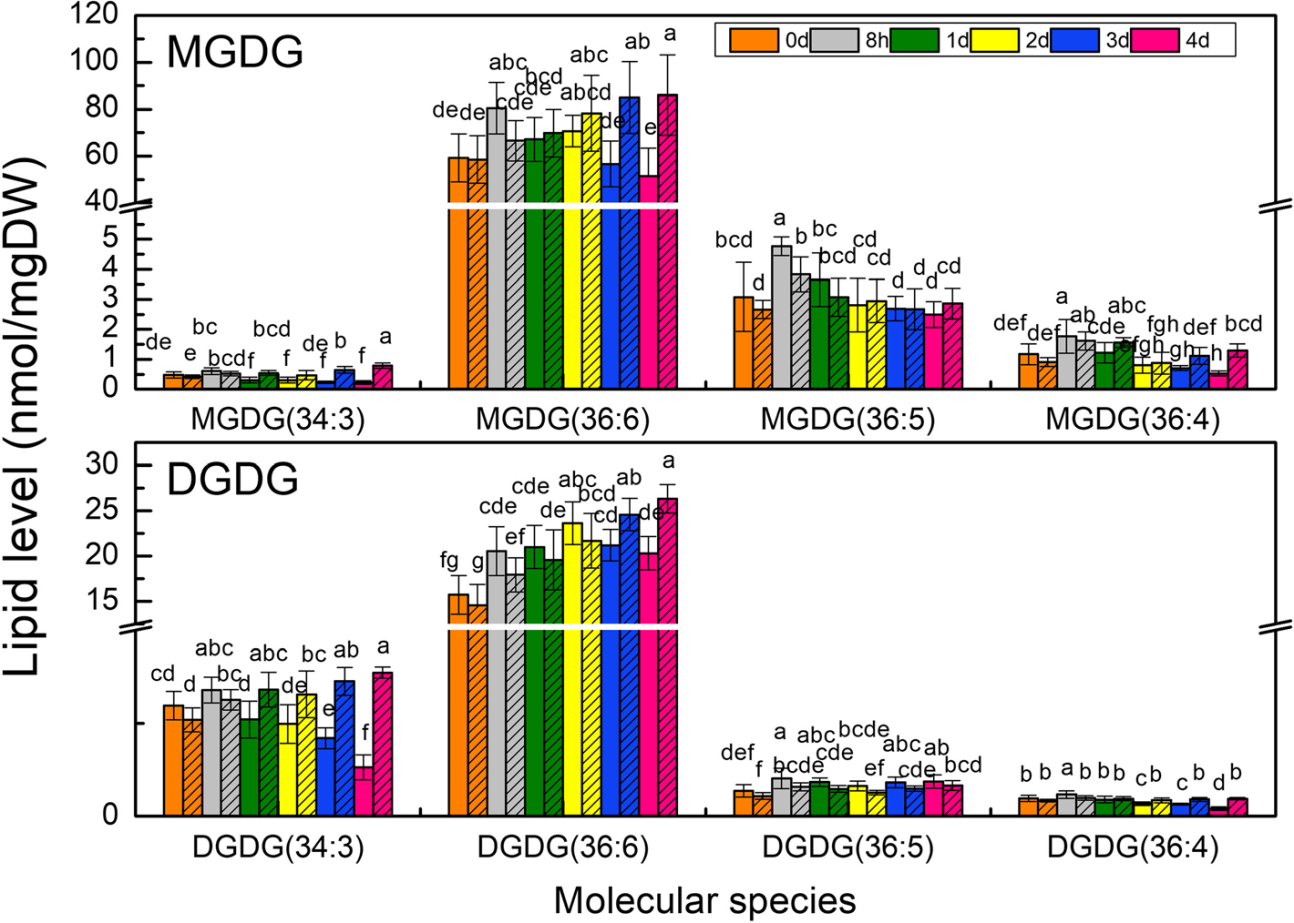
Changes in the level of the main molecular species of the two major galactolipids with water stress time. Galactolipids molecular species were determined in plants after treatment for 0 day, 8 h, 1 day, 2 days, 3 days, and 4 days under PEG (blank bars) and CK (hatched bars). Different letters indicate significant difference (*P* < 0.05). Values are means ± SE (*n* = 4 to 6)

Most molecular species of MGDG and DGDG, as well as their respective total contents in CK increased constantly with time, instead of remaining almost unchanged, indicating that these two kinds of galactolipids increase with growth of young wheat seedlings (Fig. 7; Additional file 1), thus, the change in CK should be taken into account when analyzing the variation trends of galactolipids under water stress. Still, the way in which the following interesting galactolipids responded to water stress could be made out clearly.

**Fig. 8:**
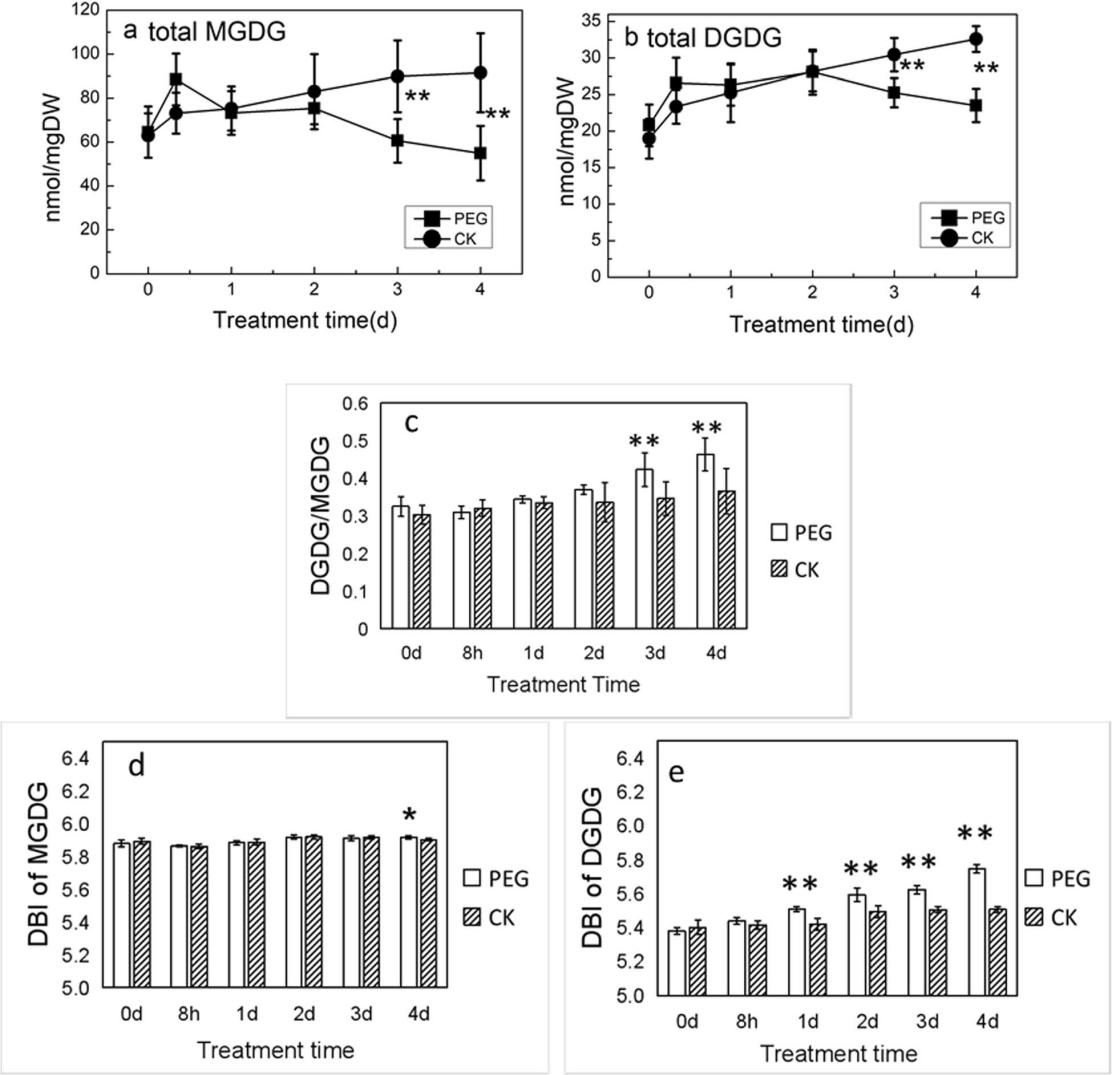
Changes of the total amount of MGDG (**a**) and DGDG (**b**) with water stress. **c** Changes in DGDG/MGDG of plants with water stress. Changes in DBI of MGDG (**d**) and DGDG (**e**) in plants with water stress. * and ** indicate significant differences between plants under treatment with PEG (PEG) and without PEG (CK) at the same time points of determination at *P* < 0.05 and *P* < 0.01 respectively. Values are means ± SE (*n* = 4 to 6)

As shown in Figs. 7 and 8a, for MGDG, the predominant molecular species MGDG (36:6) and total MGDG, declined gradually and were significantly lower in comparison with CK from 3 days after treatment, except an increase within first 8 h or so. While the minor molecular species MGDG (34:3) was decreased after 1 day of stress. On the other hand, for DGDG, the predominant molecular species DGDG (36:6), as well as total DGDG, increased in parallel with CK over time firstly, then no longer increased with CK after 3 days of stress (Figs. 7 and 8b). Minor molecular species DGDG (34:3), and DGDG (36:4) dropped after 1 day and 2 days of stress respectively, compared to CK which increased over time. DGDG (36:5) was higher than that in CK constantly. Thus, the time when MGDG (34:3) and DGDG (34:3) began to decline and the time when photosynthesis rate declined caused by non-stomatal limitation factors were almost simultaneous. And the reduction of the two predominant molecular species, MGDG (36:6) and DGDG (36:6), corresponded to the increase in membrane ion leakage.

Compared with CK, the DGDG/MGDG ratio under water stress showed a tendency to increase (Fig. 8c). The ratio became significantly higher than that in CK from 3 days after exposure to stress.

### Variations in DBI of galactolipids with water stress time

The DBI of MGDG for plants under PEG stress and without PEG kept stable during the first 3 days, while it showed significant difference after stress treatment for 4 days (Fig. 8d; Additional file 1). Though the DBI of DGDG in CK kept a stable trend, that of DGDG under PEG treatment increased with stress time proceeding (Fig. 8e). As a result, the difference between CK and stress treatment tended to enlarge with time. This indicated that the seedlings positively responded to PEG stress by modifying the unsaturation of DGDG acyl chains mainly.

### The correlation between phospholipids and membrane permeability, galactolipids and photosynthesis

**Fig. 9:**
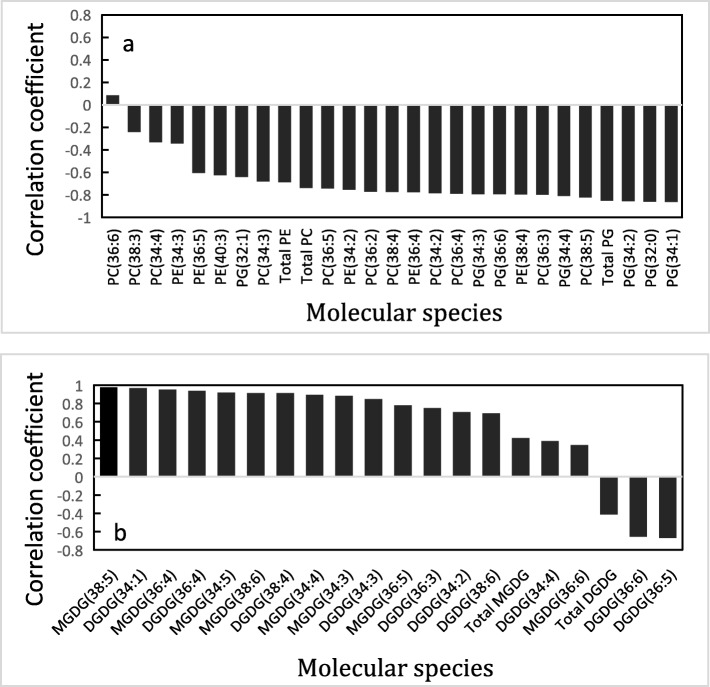
**a** The correlation between three phospholipids and membrane ion leakage based on Spearman’s correlation analysis. **b** The correlation between the alteration of photosynthesis rate and dynamic changes of galactolipids. Phospholipids profiles and membrane ion leakage, as well as galactolipids profiles and photosynthesis of leaves after PEG treatment for 0 d, 1 d, 2 d, 3 d, 4 d were analyzed by Spearman’s correlation here. All molecular species meet criteria were analyzed here (Criteria: the amount of molecular species is more than 0.0005 nmol, coefficient of variation (st. dev./average) for identical samples, made by pooling samples, is less than 0.3)

As shown in Fig. 9a and Additional file 1, most PGs, PEs, PCs showed moderate or strong negative correlation with membrane permeability (*r* < − 0.5), with the exception of PC (36:6) and PC (38:3), displaying no significant correlation with it (*r* < 0.3), as well as PC (34:4) and PE (34:3), presenting low correlation with it (− 0.5 < *r* < − 0.3). The result supported that the three classes of phospholipid contributed largely to membrane stability under water stress.

On the other hand, as shown in Fig. 9b, DGDG (34:3), MGDG (34:3) and some minor molecular species had very strong positive correlations with photosynthesis rate (*r* > 0.8). In contrast, MGDG (36:6) and DGDG (36:6) respectively showed low correlation (0.3 < *r* < 0.5) and significant negative correlation (− 0.8 < *r* < − 0.5) with photosynthesis rate. The result indicated that various molecular species of the two glactolipids might play different roles in photosynthesis.

## Discussion

In this study, a high concentration as 20% of PEG solution was used to mimic water stress for wheat seedlings. Jinmai 47, a drought resistant genotype of winter wheat, showed a significant increase in membrane permeability when suffering stress for 3–4 days. Furthermore, a dramatic membrane permeability increase, corresponding to the withering sign of young seedlings, indicated the irreversible severe damage to cellular membrane, as well as plant tissues, when stress continued for 5–6 days. Meanwhile, Pn decreased with stress time, with non-stomatal limitation turning to be a predominant limiting factor after 1 day under treatment. Lipid composition closely relates to membrane stability and fluidity, thus it is crucial in maintaining cell integrity and the activity of membrane-bound proteins [28]. Galactolipids are major components in the chloroplast envelope and thylakoid membranes, and play important roles in photosynthesis [29–33]. We adopted lipidomic analysis to comprehensively clarify how main phospholipids and galactolipids dynamically changed to discern accompanying the decline in cellular membrane stability and photosynthesis rate in the present study.

### Phospholipids degradation and membrane permeability elevation

Metabolism of membrane lipids responds to water stress differently, depending on plant species and stress intensity etc. In olive trees exposed to progressive stress for 1 month, PC, PE, and PG of leaves fell down to different degrees in both tolerant and susceptible cultivars [34]. In 2 *Linsernia* species, after dehydration stress for 3 weeks, PC, PE, PG, and PS all declined, while PI increased in desiccation tolerant *Linsernia brevidens,* declined in desiccation sensitive *Lindernia subracemosa* [3]. Similarly, the present study found general decline trends in several phospholipids in wheat seedlings suffered severe water stress induced by 20% PEG. PC, PE and PG slightly increased, then declined rapidly to a low level 2 days after stress, and kept nearly stable for the following 2 days. But PS and PI did not show a clear downward trend here. Unexpectedly, some researches, however, found increase trends in some phopholipids. A recent work conducted on two introgression genotypes of Italian ryegrass (*Lolium multiflorum*) × tall fescue (*Festuca arundinacea*) showed that PG and PE increased in both tolerant genotype 4/10 and sensitive genotype 7/6 during stress for 11 days, with genotype 4/10 increasing earlier [35]. Also, PC, PE, PS, PI increased, PG decreased in *Craterostigma plantagineum*, after desiccation for 14 days [3]. Additionally, in some species, phospholipids kept unchanged under water stress. PC, PE, and PG kept stable in *Thellungiella salsuginea*, a popular extremophile model, after water stress for 1 day and 3 days. Also, the 3 phospholipids and PS maintained unchanged in *Arabidopsis thaliana* under the same stress treatment [36]. The mechanisms underlying the different responses of lipid composition in various plants under water stress remain to be deeply understood.

Membrane phospholipids are mainly hydrolyzed by phospholipase D (PLD), a major family of lipid hydrolyzing enzymes in plants, which can be activated by a wide range of stresses [37]. PLDs preferentially catalyze hydrolysis of phospholipids, generating PA and a free head group. Pappan [38] reported the in vitro substrates of PLD α, β, γ were PC, PE, PG, and PS, but none of the three PLDs used PI as substrates. Zien [39] and Welti [2] found that the major substrate in vivo of the most abundant phospholipase D, PLDα, was PC, rather than PE and PG. Activities of these phospholipases lead to alterations in absolute content and proportion of lipids. Lipidomic profiling in this study showed the levels of the main molecular species of PC, PE, and PG, as well as their total contents declined largely to the low levels within 2 days, indicating that PC, PE and PG were the primary degradation targets in wheat leaves under PEG-induced water stress. Among the 3 phospholipids, PC was degraded in largest degree. Also, the ratio of PC to PE decreased to lower level after 2 days of stress, implying that PC was preferentially hydrolyzed rather than PE, or higher PC-to-PE conversion.

Phospholipids play roles in membrane stability by means of structure alteration. Russel [40] found that non-lamellar domain in the plasma membrane caused interruption of the bilayer structure and hence rendered high permeability. PC, PG and PS prefer to form stable bilayer structure. However, PE has great tendencies to form a non-lamellar structure, hexagonal phase II, which is less stable [2, 6, 41]. Therefore, rapid drops in PC, PG under water stress rendered the membrane unstable, and the ion permeability of cellular membrane increased as a result.

These lipids declined to low levels earlier than the membrane ion leakage dramatically increased, implying that the loss of membrane integrity lagged behind severe membrane phospholipid degradation. A slight increase in absolute contents of the main molecular species of these lipids occurred at an early stage of stress, as observed 8 h after exposed to stress. The stress severity depended on stress time in our experiment. This might imply that membrane lipid synthesis increased to positively resist mild water stress at early stage, and degradation under severe stress at late stages.

### Galactolipid degradation and photosynthetic rate reduction

In this study, water stress caused the gradual decrease of total MGDG except an increase within first 8 h or so, while total DGDG increased firstly, and decreased after 2 days or so. Moreover, DGDG/MGDG increased after 3 days under stress. The results indicated relatively higher accumulation of DGDG, higher degradation of MGDG, or the conversion from MGDG to DGDG. DGDG can be produced by two pathways, (1) By UDP-Gal-dependent DGDG synthases (DGD1, DGD2) [42, 43], and (2) By SENSITIVE TO FREEZING 2 (SFR2), which transfers the galactose head group from MGDG to form DGDG and oligogalactolipids (OGDG) in the plastid envelope membrane [27]. SFR2 is required for freezing tolerance in cold-acclimated conditions presumably via stabilizing the chloroplast membrane [44]. Regarding to water stress, Chen suggested that higher expression in DGD pathway was responsible for DGDG accumulation according to higher expressions of DGD and MGD genes [25]. In desiccated *C. plantagineum*, the outermost galactose (GalII) in DGD was in the α-configuration, indicating it was synthesized by DGD pathway [3]. Thus it was probably the activation of DGD pathway by water stress that led to the increase in DGDG and DGDG/MGDG in Jinmai 47, which needs further studies to prove.

MGDG is more likely to form unstable hexagonal phase II due to its conical shape with a small galactose head group and flexible poly-unsaturated fatty acid tails [21, 22, 44]. In comparison, DGDG is apt to form stable bilayer lamellar phase easily, due to the bulky head group with two galactoses, which lay a foundation for its cylindrical shape. The elevation of DGDG/MGDG might be a common strategy for plants to adapt to adversities by maintaining a physical state which supports normal function of membrane proteins, and participating in maintaining the bilayer membrane structure [45, 46]. This was confirmed by a number of recent studies in different plants, such as *Thellungiella salsuginea* [36], cowpea [7], maize [25], and soybean (*Glycine max cv* Jack) [47]. In this study, the material utilized was Jinmai 47, a drought resistant wheat cultivar. DGDG/MGDG increased gradually with stress time, and was significantly higher than that of CK after 3 days under PEG treatment. This might imply that wheat leaves adapted to water stress by regulating the ratio of DGDG/MGDG to maintain the stability of chloroplast envelope and thylakoid membrane.

On the other hand, galactolipids are involved in photosynthesis light reaction [26, 27]. Recent studies reported that MGDG and DGDG existed in PS I, PS II, and Cyt b6f complex [48–50]. Several investigations applied genetic manipulation to prove the direct role that MGDG played in photosynthesis. MGDG deficiency in knockdown mutant mgd1 in tobacco led to decreased cytochrome b6f complex and electron transport of PSII apparatus [51]. An Arabidopsis mutant, *amiR-MGD1*, had a lower level of MGDG comparing with wild type, and the activity of PSII and the energy coupling between reaction and antenna complexes were impaired [52]. Because of the direct involvement in photosynthesis, degradation in MGDG and DGDG may inevitably result in reduction in Pn promptly. In the present study, MGDG (34:3) and DGDG (34:3) and some minor molecular species decreased promptly, approximating to the time when dramatic reduction in Pn occurred due to non-stomatal limitation mainly. The two predominant molecular species MGDG (36:6) and DGDG (36:6), however, declined later than the time when non-stomatal limitation factor began to exert. Thus we speculate that MGDG (34:3), DGDG (34:3) and some minor molecular species might be associated with key components of photosynthetic protein-cofactor complexes, and participate in photosynthesis directly. The two predominant molecular species might locate in the balk lipid of the thylakoid lipid bilayer matrix and play their roles as stabilizing the membrane when encountering adverse situations. The speculation was also supported by results of Spearman’s correlation analysis as shown in Fig. 8e. Direct evidence needs to be obtained by further studies on the intracellular distribution patterns of lipid molecular species. If obtained, such evidence would strongly support the notion that different molecular species play distinct roles in physiological process.

### Modification of lipid unsaturation under water stress

Previous studies surmise that the unsaturation degree of fatty acids changed in order to prevent membrane lipids from phase transition, thus improving stress resistance under adverse environment. In *Arabidopsis thaliana*, DBI of membrane lipids was found to be increased to tolerate low temperature [2, 17], while decreasing to resist high temperature [53, 54]. Under water stress, lipid unsaturation remained constant or increased in spinach (*Spinacia oleracea* L.) [55], coconut palm (*Cocos nucifera* L.) [10], and olive tree leaves (*Olea europaea* L.) [34]. In our study, lipidomic analysis revealed that lipids differed in regulating unsaturation degree under water stress. DBI of PE and MGDG remained unchanged. PC began to modulate DBI after 2 days under severe stresses. DGDG began to modify DBI promptly, as observed on the first day of stress. While DBI of PG, PI and PS were lower in different time under stress than that of CK. PC and DGDG might be involved in mediating the response to water stress by increasing unsaturation.

## Conclusion

Under PEG-induced water stress, membrane ion leakage significantly increased after 3 days treatment, and dramatically increased after 4 days. Meanwhile, the molecular species of PC, PE, and PG were largely degraded within 2 days. The collapse of integrity and stability of cellular membranes lagged behind the serious degradation of PC, PE, and PG. None of the main molecular species of PS showed a clear downward trend. Thus PS was not the main phospholipid degraded under water stress. MGDG (34:3), DGDG (34:3) decreased approaching the time when the photosynthesis rate decreased mainly due to non-stomatal factors. On the other hand, two predominant galactolipid molecular species, MGDG (36:6) and DGDG (36:6), significantly decreased at later time. Thus, we speculate that different galactolipid molecular species might have distinct functions. MGDG (36:6) and DGDG (36:6) might mainly play their roles in stabilizing the membrane system under adverse situations, while MGDG (34:3) and DGDG (34:3) might not only establish the lipid bilayer of photosynthetic membranes, but also exist in photosynthetic protein-cofactor complex and participate in photosynthesis directly. Moreover, the DBI of DGDG and PC ratcheted up with water stress, and was likely to be part of a strategy for Jinmai 47 to maintain membrane stability. To obtain direct evidence, further studies on the intracellular distribution patterns of galactolipid molecular species need to be carried out.

## Methods

### Materials and growth conditions

Plant materials applied was Jinmai 47, a well-known water-resistant cultivar, which is planted widely in the rain-fed area of the North China Plain. The wheat seeds we utilized were provided by Dryland Wheat Breeding Group, Cotton Research Institute, Shanxi Academy of Agricultural Sciences, Yuncheng, Shanxi Province, China. The growth conditions of wheat were described in our previous study [56]. Minor alterations were made. We soaked surface-sterilized wheat seeds in distilled water for 8 h, and placed them upon water-wetted filter papers in culture dishes to germinate. The experiment was conducted in a growth chamber with 16 h /8 h photoperiod under light intensity of 300 μmol m^− 2^ s^− 1^ at day/night temperature of 23 °C/20 °C. Seeds germinated in dark condition for 2 days, seedlings then grow up in distilled water for 5 days, and were transplanted in pots with 1/2 Hoagland nutrient solution for 8 days. Similar seedlings were selected and randomly assigned to two treatments, a control (CK) and a water stress treatment [56]. Plants were cultivated in 1/2 Hoagland nutrient solution continuously in CK, and were cultivated in 1/2 Hoagland nutrient solution with 20% polyethylene glycol (PEG) in the water stress treatment. For each treatment, 6 replicates for lipidomics analysis, 3 for membrane ion leakage determination, and 3 for photosynthetic parameters measurement were arranged, respectively. For lipidomics analysis and leakage determination, every replicate includes 5 leaves. For photosynthesis, each replicate includes 2 leaves.

### Measurement of lipidomics of leaves

#### Extraction of lipids in leaves

The second fully expanded leaves of wheat seedlings treated by 20% PEG and CK for 0 day, 8 h, 1 day, 2 days, 3 days, 4 days were cut and immersed in glass screw-cap (Teflon-lined) tubes, 3 ml isopropanol with 0.01% butylated hydroxytoluene (BHT) preheated at 75 °C for 15 min were added in order to terminate the lipolytic activities. 1.5 ml of chloroform and 0.6 ml of water were added into the tubes, and they were vortexed and agitated at room temperature for 1 h. Pasteur pipettes were used to transfer lipid extracts to new glass screw-cap (Teflon-lined) tubes. For the remaining leaf material, 4.0 ml of chloroform/methanol (2:1, v/v) with 0.01% BHT were added in, and shaked for 60 min. This extraction procedure was repeated for 7 times on each sample until the leaves of every sample become white and the following extracts were combined with the first extract. 6 replicates were measured per treatment.

The extracts were washed once with 1.0 ml of 1 M KCl and once with 2.0 ml of water, vortexed and centrifuged to break the phases, and the upper phase was discarded. A nitrogen evaporator (Organomation Associate, Inc. Berlin, MA, USA) was used to evaporate solvents completely. After that, extracts were redissolved in 1.0 ml of chloroform and transferred to 2.0 ml clear glass vial with Teflon-lined screw cap. Before shipping, the solvent was evaporated completely again. At the end, we dried the extracted leaves at 105 °C oven overnight and determined the dry weight using a balance (QUINTIX125D-1CN, Sartorius, accurate to 0.00001). The methodology of lipid extraction from leaves was described by Welti with minor modifications [2, 57].

### ESI-MS/MS analysis of lipid molecular species and data processing

ESI-MS/MS analysis was carried out by Kansas Lipidomics Research Center. The lipid molecular species were identified by precursor or neutral loss scanning, and the lipids in each head group class were quantified in comparison with internal standards. The internal standards were described in Xiao [58]. The amounts of internal standards used were: 0.6 nmol PC (12:0), 0.6 nmol PC (di 24:1), 0.3 nmol PE (di12:0), 0.3 nmol PE (di20:0 (phytanoyl)), 0.3 nmol PE (di 23:0), 0.3 nmol PG (di 14:0), 0.3 nmol PG (di 20:0 (phytanoyl)), 0.287 nmol PI (16:0–18:0), 0.105 nmol PI (di18:0), 0.2 nmol PS (di 14:0), 0.2 nmol PS (di Phy), 0.44 nmol DGDG (16:0–18:0), 1.48 nmol DGDG (di18:0), 1.665 nmol MGDG (16:0–18:0), and 1.405 nmol MGDG (di18:0). Samples were analyzed on a triple quadrupole MS/MS (4000QTrap, Applied Biosystems, Foster City, CA), and data processing were conducted as described in Xiao [58]. Sequential precursor and neutral loss scans of the extracts produce a series of spectra with each spectrum revealing a set of lipid species containing a common head group fragment. Lipid species were detected with the different scans, the collision energies, the entrance potentials, and the exit potentials were different for each lipid. The lipids in each class were quantified in comparison to the internal standards of that class. The produced data were in the units nmol/mg finally.

### Calculation of lipid double bond index (DBI)

Referring to Rawyler [59], DBI = Σ(n × mol% lipid)/100, where N is the total number of double bonds in the two fatty acid chains of each glycerolpid molecule.

### Membrane permeability measurements

The method of the measurement of membrane permeability referred to [60]. Some minor alterations were made here. We detached the second fully expanded leaves from seedlings cultivated in both treatments for 0 day, 1 day, 2 days, 3 days, 4 days, 5 days, 6 days. Leaves were washed briefly with deionized water, and were cut into 1 cm fragments and immersed in 10 ml of deionized water. After that, a pump was used to exhaust air from leaves for 30 min, leaves were agitated for 3 h followed by. We measured and recorded the first conductivity. The second conductivity (total conductivity) was measured by boiling the leaf fragments in bathing solution for 15 min. Three replicates were measured per treatment [56].

$$ \mathrm{Relative}\ \mathrm{Conductivity}=\frac{1\mathrm{st}\ \mathrm{Conductivity}}{2\mathrm{nd}\ \mathrm{Conductivity}}\times 100\% $$

### Photosynthesis measurements

The gas exchange parameters, including photosynthesis rate (Pn), intracellular CO_2_ concentration (Ci), stomatal conductance (Gs), of the second fully expanded leaves treated for 0 h, 1 d, 2 d, 3 d, 4 d were measured by a photosynthesis system (Li-6400; LI-COR Inc., Lincoln, NE, USA) between 9:00 a.m-11:00 a.m. A standard 2*3 cm chamber and light-emitting diode light source was used to support constant photosynthetically active radiation level of 1000 μ mol ⋅ m^−2^s^−1^. All measurements were taken at a CO_2_ concentration of 400 μ mol ⋅ m^−1^. Two leaves were measured and averaged as the value for each replicate. Stomatal limitation index (Ls) = 1-Ci/Ca, Ca = 400 μmol·mol^− 1^ here; non-stomatal limitation index = Ci/G_s_ [61, 62]_._

### Statistical analysis

Data were analyzed by SAS statistics software. One-way ANOVA was conducted on all lipidome data. The statistical significance was tested by the Duncan and Tukey method. T-test was conducted on the data from membrane ion leakage and photosynthesis parameters. Correlation analysis was conducted on phospholipids and membrane ion leakage, and photosynthesis rate and galactolipids.

## Supplementary information

**Additional file 1.** Data of glycerolipids, photosynthesis parameters, membrane ion leakage, DBI of wheat leaves during water stress, and the correlation between membrane ion leakage and phospholipids, photosynthesis rate and galactolipids.

## Data Availability

The datasets used and/or analyzed during the current study are available from the corresponding author on reasonable request.

## Abbreviations

Ci: Intracellular CO_2_ concentration
Ci/Gs: Non-stomatal limitation index
DBI: Double bond index
DGDG: Digalactosyldiacylglycerol
G_s_: Stomatal conductance
MGDG: Monogalactosyldiacylglycerol
PC: Phosphatidylcholine
PE: Phosphatidylethanolamine
PEG: Polyethylene glycol-6000
PG: Phosphatidylglycerol
Pn: Net photosynthetic rate
